# Bariatric surgery and exercise: A pilot study on postural stability in obese individuals

**DOI:** 10.1371/journal.pone.0262651

**Published:** 2022-01-14

**Authors:** Natálie Cibulková, Klára Daďová, Kateřina Mašková, Andrew Busch, Alena Kobesová, Jitka Vařeková, Marcela Hašpicová, Martin Matoulek

**Affiliations:** 1 Faculty of Physical Education and Sport, Charles University, Prague, Czech Republic; 2 Third Department of Internal Medicine, General University Hospital in Prague, Prague, Czech Republic; 3 Health and Human Kinetics, Ohio Wesleyan University, Delaware, Ohio, United States of America; 4 Department of Rehabilitation and Sports Medicine, 2nd Faculty of Medicine, Charles University and University Hospital Motol, Prague, Czech Republic; 5 Medical center ORP centrum, Prague, Czech Republic; University of Catanzaro: Universita degli Studi Magna Graecia di Catanzaro, ITALY

## Abstract

This study aimed to analyze the changes in postural stability of individuals with obesity after bariatric surgery, and the effect of three months of regular exercise on the static postural stability. Twenty-two subjects (7 females and 15 males) aged 31 to 68 years (Body mass index 35–55 kg.m^-2^) completed the study. Participants were divided into two groups: one group participated in an exercise program after the bariatric surgery (n = 10; age 48.9 ± 7.5 years; Body mass index 42 ± 5.6 kg.m^-2^) while the second group did not exercise at all after bariatric surgery (n = 12; age 44.7 ± 13.6 years, Body mass index 42.6 ± 6.0 kg.m^-2^). Static postural stability was measured using a Tekscan MobileMat pressure plate before and 4 months after the bariatric surgery. The exercise program included exercising three times a week including: one hour of strengthening, one hour of aerobic group exercise and at least one session of individual exercise at home. There were no significant differences in Center of force sway, Center of force ranges and average speed before and 4 months after bariatric surgery. Also, no effect of exercise was found. Post-pre differences of some parameters were negatively related to age (r from—0.46 to—0.72). Further studies are needed to explore this topic in depth.

## Introduction

Obesity has become a major global health concern that is gaining prominence not only in health care, but also in the economy, where one of the frequent topics are the economic consequences of obesity (e.g. lower wages, a lower probability of employment, and higher medical care costs) [1]. Growing evidence also indicates that being overweight negatively affects functional aspects such as standing up [2], walking [2], balance and stability [3].

Postural stability is often described as the ability to respond to changes in internal and external forces and the ability to ensure an upright posture to prevent falls [4, 5].

Many studies report worse static and dynamic postural stability in individuals with obesity compared to individuals with normal body mass index (BMI) values [6–11]. However, there are also studies that oppose the above outcome [12, 13].

There are many factors that determine postural sway and postural stability, where anthropometry of the body (height and weight) appears to be one of the most supported [14]. Weight gain with abnormal or excessive fat accumulation is associated with changes in geometry and posture [15, 16]. Increased adiposity and insufficient musculature in obese individuals may alter bipedal stance and gait, reduce musculoskeletal tissue quality, and impair neuromuscular feedback. These physiological changes have an impact on stability [17].

In the treatment of obesity, dietary measures, regulation of physical activity, psychotherapy, pharmacotherapy, and balneology methods are most often used. Bariatric-metabolic surgical treatment of obesity is usually considered in patients with a BMI ≥ 40 kg.m^-2^ or in patients with a BMI ≥ 35 kg.m^-2^, if other associated diseases are present, such as hypertension, type 2 diabetes mellitus, ischemic heart disease etc. [18].

However, effects of rapid weight loss after bariatric surgery, especially on the musculoskeletal system, motor control and postural stability, are not fully known [19].

Compared to subjects with normal weight, individuals with obesity have a different distribution of mass and thus a different center of gravity, which is especially important when changing weight. Frequent loss of muscle mass after bariatric surgery could also compromise stability due to functional and physiological changes in musculoskeletal composition, center of gravity and coordination [20].

As mentioned above, an integral part in the treatment of obesity is the inclusion of appropriate physical activity, which increases energy expenditure and leads to a reduction in abdominal fat as well as cardiac and metabolic risks [21]. There are studies examining the effect of exercise programs on improving postural stability in individuals with obesity [8, 22, 23]. However, the results of these studies show that the exercise programs do not always have a significant effect on the change of static and dynamic stability. Therefore the aim of this study was to determine the effect of three months of regular exercise, including strength and aerobic exercise, on static postural stability in individuals with obesity who have undergone bariatric surgery.

## Materials and methods

### Design

The study was a longitudinal observational study assessing postural stability before and after bariatric surgery (BS), and the effects of a 3 month structured exercise program. Participation in the exercise group was voluntary, as they agreed to adhere to the exercise protocol for the allotted 3 month time period.

### Participants

Twenty-two patients (7 females, 15 males; age = 31–68 years; BMI = 35–55 kg.m^-2^, obesity grade 2 and 3) who underwent BS volunteered to complete this study. This convenience sample included patients with long-term obesity (throughout their adult life) treated at the 3rd Department of internal medicine at the General University Hospital in Prague dating from 2018–2020. Inclusion criteria required the presence of grade 2 or 3 obesity and a planned bariatric surgery within the age range of 30–70 years. A total of 107 patients were initially recruited for eligibility to participate. Eighty-five patients were excluded from the study due to a combination of the following: failure to undergo planned bariatric surgery for various reasons, self-termination of treatment, inability to attend examinations during the testing period, disease occurrence preventing complete examination, or diagnosis of diabetes mellitus. Patients were also excluded if they presented with impaired stability control components (ataxia, stroke, Parkinson disease etc.), sensory components (uncompensated visual disturbances, vestibular apparatus, neuropathy) or performance components (e.g. paresis, significant injuries, limb surgery etc.), or if they exercised regularly more than three times per week for 45–60 minutes. The type of surgery was chosen according to medical history and prognosis of the patient, which included gastric plication (n = 15), sleeve gastrectomy (n = 4), and gastric bypass (n = 3). Group assignment was based on participants’ self-selection into the exercise program. Demographic characteristics of both groups are listed in Table 1. This study was approved by the Ethics Committee of Charles University, Faculty of Physical Education and Sport (protocol code 234/2017, approved July 25, 2018), and written informed consent was obtained from all participants before beginning the study.

**Table 1:** Demographic data of the study groups at baseline.

	Exercising group (EX)	Non-exercising group (NEX)	p-Value
Number [n (women; men)]	10 (4;6)	12 (4;8)	
Age (years)	48.9 ± 7.5	44.7 ± 13.6	0.389
Height (m)	1.75 ± 0.13	1.75 ± 0.10	0.847
Weight (kg)	128 ± 23.1	131 ± 22.8	0.737
BMI (kg.m^-2^)	42 ± 5.6	42.6 ± 6.0	0.825

Note: Values are expressed as means± SD.

### Postural stability evaluation

A portable MobileMat^™^3140 measuring board (TekScan Inc., South Boston, Massachusetts, USA) with standard resolution and pressure mapping technology which included extensive research and development tools was used. It was connected to a computer where data were processed via USB cable using SportsAT software [24]. The measuring plate has overall dimensions of 63.6 x 55.9 x 4.2 cm. The sensing area is 48.7 x 44.7 cm and the height of the platform in the sensing area is 0.76 cm. Scanning speed is up to 185 Hz, depending on the resolution. One pressure sensor for every cm^2^ is built into the board and the range of measurable pressures is 345 to 862 kPa. The total weight of the board is 3.5 kg [24, 25]. The MobileMat board has an established reliability and validity for measurement of postural stability [26–28] and used for research in cohorts with ranging BMI [29, 30].

The initial measurement was performed approximately 1 to 2 weeks before the bariatric surgery, and the output measurement was performed in both groups 4 months after the surgery. The measurement took place in a calm and quiet environment, the board was placed on hard ground 2 meters from the wall. The participants were examined in underwear, barefoot and their natural width of the standing base. All participants were measured with their eyes open (OE) and with their eyes closed (CE), each measurement lasting 30 s, so in total the measurement took 1 minute.

The participants did not perform practical trials prior to postural stability assessment. The participant first stood on the MobileMat board and was asked to stand as still as possible, looking at the wall in front of him/her at one place at eye level. The measurement was then started with the eyes open for 30 s. Subsequently, the participant was asked to close his/her eyes and to try standing as still as possible. When he/she was ready, the measurement was turned on again for 30 s.

Four postural stability parameters with OE and CE were evaluated: the total length of the COF sway, length of antero-posterior and medio-lateral COF range, and COF average speed. For all above-mentioned parameters, a higher value indicates worse stability, while a lower value indicates better stability.

### Exercise program

The main purpose of the exercise program was to maintain lean body mass and improve functional abilities. Improving stability was not the primary focus of the exercise program, rather part of it. Precise nutritional assessment was provided by nutritional therapists for all patients, in which a low-calorie diet was prescribed, which is commonly indicated for patients after BS.

The exercise program was applied one month after the bariatric surgery and lasted 12 weeks (three months). It included exercising three times a week: one hour of group strength training once a week, an hour of aerobic group exercise once a week, and independent home fitness session at least once a week. Aerobic and strength training took place in the Reconditioning Center of VŠTJ Medicina Prague under the guidance of a physiotherapist or a trained instructor. Patients recorded the exercise online in the Time for Health application (a tool for monitoring the diet and physical activity of patients) or in paper form in the activity diary.

The exercise program was designed by a sport scientist and physiotherapist. The exercise program was adapted to the capabilities and overall condition of each participant. Each strength training unit had the same layout as shown in Table 2. The unit consisted of a whole warm-up using aerobic machines for 10–15 minutes followed by a preparation section of approximately 10 minutes. This was followed by a strengthening part in the form of circuit training to strengthen all major muscle groups. In this part, there were three repetitive series of exercises. There were always 7–10 exercises of one minute in each series, so a total of one series lasted approximately 10 minutes and there was always a short pause between the individual series. The difficulty of the exercises was chosen on the basis of the initial examination, when it was adapted to each patient, for example, by choosing an alternative exercise with lower or higher difficulty. Intensity was chosen according to the current level of fitness. It was guided by subjective evaluation of the training using the Borg scale, maintaining values between 12 and 13. Current pain, fatigue and shortness of breath were also taken into account when adjusting the intensity. Between each exercise, a break was needed only to move to the next station (10 s). Exercises with their own body weight, dumbbells, fitness machines, medicine balls, resistance bands, unstable surface training were used. In total, the main part of the training took 30–35 minutes. This was always followed by a final soothing exercise with mainly stretching exercises and breathing for approximately 10–15 minutes. The total training unit duration was 60 to 75 minutes.

**Table 2:** Description of the strengthening exercise unit parts.

Training Segment	Length (min)	Description
Warm-up	10–15	Total warming up and preparation using aerobic devices
Preparation	10	Preparation of muscle and joint structures using circular and pendulum movements, respiratory physiotherapy to prepare the respiratory system for exercise
Resistance Exercises	30–35	Strengthening the muscles of the whole body, training stability, stabilization, support
Cool-down	10–15	Soothing exercises with mainly stretching exercises and respiratory exercises to calm down

Aerobic training was carried out using fitness machines equipment. Participants could choose, according to their own choice based on the current state of health, from a cycle ergometer, a treadmill, a rowing machine, an elliptical trainer, or an ergometer which was used for the upper half of the body. One aerobic exercise session took 60 minutes depending on the condition of the participants; and the participants could alternate between different devices. The intensity of the exercise was set at a level of 12–13 according to the Borg scale.

The individual home-based exercise sessions consisted of a series of seven simple exercises (14 repetitions of 2 series). The total training unit duration was 30 to 40 minutes and was performed once a week. The participants performed the home-based exercise on days other than days when circular or aerobic training took place. The participants recorded the exercises in the web application or in their own activity diary.

### Data analysis

The data were first visually processed by SportsAT software and the data from individual measurements were then entered into a Microsoft Excel 2010 spreadsheet. The Microsoft Excel statistical analysis tool as well as STATISTICA (StatSoft, Tulsa, OK, USA, version Cz. 13) were used to process the data. Means and standard deviations (SD) were calculated for each continual variable. After checking data for normality and homoscedasticity, it was decided to use non-parametric statistics methods (Mann-Whitney U test, Wilcoxon Signed Rank Test, Friedmann ANOVA, Spearman Rank Correlation). Statistical significance was determined *a priori* at *p* < 0.05.

## Results

### Postural stability and anthropometric data of all subjects before and 4 months after BS

A total of 4 parameters of postural stability with both eyes open and closed were evaluated: total length of the COF sway, COF antero-posterior (AP) and medio-lateral (ML) range and COF average speed. Table 3 shows the mean values of all these parameters with open and closed eyes (OE / CE) as well as weight and BMI in the entire sample before BS and 4 months after BS.

**Table 3:** Mean values (±SD) of weight, BMI, and postural stability parameters in all subjects (N = 22) before and 4 months after bariatric surgery (within group comparison).

		Before BS	After BS	p-Value
**Weight (kg)**		131.4±22.5	113.2±19.8	<0.001
**BMI (kg.m** ^**-2**^ **)**		42.6±6.0	36.7±4.9	<0.001
**COF sway (cm)**	OE	28.28±10.96	27.89±10.74	0.684
	CE	45.70±18.75	47.55±23.25	0.372
**Range AP (cm)**	OE	1.96±0.79	2.18±0.74	0.108
	CE	2.89±0.83	2.93±1.07	0.961
**Range ML (cm)**	OE	1.76±0.94	1.87±0.80	0.498
	CE	2.06±1.21	2.31±1.36	0.306
**COF speed (cm/s)**	OE	0.94±0.37	0.93±0.36	0.697
	CE	1.53±0.63	1.59±0.78	0.372

Note: COF—center of force, OE—open eyes, CE—closed eyes, SD—standard deviation.

In preliminary analyses comparing all subjects before and after BS, a statistically significant difference in weight and BMI were noted. There were no significant differences noted in the postural stability (PS) parameters, as many evidenced a tendency to worsen. As for gender differences, there was a statistically significant difference between men and women before BS in the CE setting for COF sway and COF speed (p <0.05). No differences were noted according to surgery type.

### Between-group comparison of postural stability and anthropometric data

In both groups there was a statistically significant weight loss (p <0.001) 4 months after the surgery. In the intervention group (EX), the average weight loss was 19.68 ± 7.41 kg and in the group of bariatric patients doing no exercise (NEX) the average weight loss was 16.95 ± 5.13 kg. However, the difference between groups was not statistically significant.

Table 4 shows the post-pre differences in the two groups in all postural stability parameters with open and closed eyes (OE / CE) as well as in BMI. There were no differences between Intervention (EX) and Control (NEX) group. It was also confirmed by Kruskal-Wallis ANOVA by Ranks.

**Table 4:** Absolute post-pre differences in BMI and postural stability parameters in the intervention and control group (between groups comparison).

Post-pre difference (mean ± SD)		Control (NEX)	Intervention (EX)	p-Value
**BMI (kg.m** ^**-2**^ **)**		-5.46	-6.58	0.621
**COF sway (cm)**	OE	-0.65	-0.76	0.717
	CE	-0.23	1.90	0.869
**Range AP (cm)**	OE	0.09	0.27	0.717
	CE	-0.21	0.32	0.199
**Range ML (cm)**	OE	-0.08	0.16	0.598
	CE	0.24	0.07	0.575
**COF speed (cm/s)**	OE	-0.02	-0.02	0.742
	CE	-0.01	0.06	0.869

Note: COF—center of force, OE—open eyes, CE—closed eyes, SD—standard deviation.

### Relationship of postural stability, height, BMI, weight changes and age

We also investigated possible relationships between measured variables. There was a significant relationship between height and initial COF sway (r = 0.57, p <0.05; see S1 Graph) and COF speed (r = 0.57, p <0.05) in closed eyes setting. This relationship decreased and became non-significant in the post-measurement. BMI values correlated positively with the COF sway post-pre difference in the open eyes setting (r = 0.44 for BMI before surgery, see S2 Graph, and r = 0.45 for BMI after the surgery, p <0.05). Similarly, both BMI before and BMI after the surgery correlated with post-pre differences in Range AP (r = 0.46 resp. r = 0.49, p <0.05) and COF speed (r = 0.45 resp. r = 0.48, p <0.05), again in open eyes setting. Weight difference was only related to age (r = 0.49; p <0.05). The initial postural stability—the total length of the COF sway with open eyes (COF sway-OE) was positively related to age (r = 0.60; p <0.05). The absolute post-pre difference in COF sway-OE was negatively related to age (r = -0.46; p <0.05). The age also related to COF range ML-OE difference (r = -0.72; p <0.001; see S3 Graph) and speed-OE (r = -0.48; p <0.05).

## Discussion

The aim of this study was to evaluate the effect of a three-month exercise program on postural stability in obese individuals undergoing bariatric surgery. A total of 4 parameters of static postural stability were evaluated using data obtained from a portable pressure plate with open and closed eyes. However, no improvement was observed in all subjects after BS including the exercise group. To our knowledge, this is the first study attempting to investigate the effectiveness of a cardio/strength exercise program (commonly recommended for obese individuals [31]) on static postural stability in BS patients using static platform posturography. Only three earlier studies have addressed the topic of evaluating static postural stability using stabilometric platforms in bariatric patients [15, 19, 32], and none of which examined the effect of exercise on static postural stability. One prior study examined an exercise program on postural stability in BS patients, but used functional testing to measure postural stability [22].

Postural stability can be evaluated, depending on the test system used, according to the center of pressure (COP), center of balance (COB) or center of force (COF) [23]. Our study is the first to use a pressure plate for testing postural stability after bariatric surgery and not a force plate (where the output is COP). COF and COP values while measuring static postural stability are comparable. For this reason, our COF results, arising from the pressure plate testing, are further compared with the COP parameter.

Measuring the rate of COP deflection is one of the most widely used parameters to evaluate static postural stability using a stabilometric plate, and the total velocity values or the average velocity values in a particular direction have traditionally been used [33]. Some studies have even shown that this parameter is the most reliable and gives the most information about postural stability [34].

Our results are in agreement with that of Benetti et al. [19], where no differences were observed in the displacement area or velocity from the center of pressure in the mediolateral and anteroposterior directions which were evaluated using a force platform in 16 patients who underwent bariatric surgery. The three-month exercise program and rapid weight loss noted in our study did not interfere with the total length of the COF sway; either in the anteroposterior and mediolateral directions, nor in the speed of COF. Therefore, these parameters are more likely to depend on the integration of the entire neuromuscular system to maintain postural stability and may be more related to learning than to body weight. This effect is probably due to the long adaptation period of overweight individuals [19].

The unchanged amount of COF displacement may be due to inadequate time for the proprioceptive system to adjust to the new body weight. Massive weight loss (MWL) and higher proportional lean mass can modify a person’s center of mass, and therefore COF displacement parameters. This can also lead to lower extremity deformities (lipodystrophy or increased gynoid contour) that could theoretically impact the COF displacement as well [35]. Accordingly, adjustments to spatial parameters in static postural stability are more neuromuscular than mechanical, and more time may be required to adjust and observe improvements [19]. In the study by Alonso et al. [36], a population study without restrictions on body weight or BMI, static postural stability as measured by posturography was only slightly influenced by the anthropometric variables. However, Hue et al. [7] evaluated 59 male subjects using a force platform and reported that an increase in body weight correlated with higher static postural instability. We assumed a change in the parameters evaluating static postural stability with weight reduction accompanying bariatric surgery, but probably the displacement from the COF was still small to detect the change. Alternatively, the change in weight was not large enough to change the COF. It would also be appropriate to evaluate lean body mass and body fat mass. The combination of aerobic exercise with resistance training is the prevention of weight gain with some positive effects on anthropometric indices, such as reduced fat mass and stable or improved lean body mass [37, 38].

The patient’s adaptation to the new body weight and the lack of specific training may explain the insufficient improvement in postural stability assessed by displacement (range) from COF. These data agree with the findings of Bankoff et al. [39], which state that heavier bodies are difficult to unbalance, but weight does not affect the displacement speed from COP. Ferreira [40] stated that the body weight interferes with the position (height) from the COP. Gaining or losing of body weight may affect the height from COP as well as body mass distribution. Furthermore, distribution of mass in the body is also affected by gender.

Several authors claim that a reduced proprioceptive response can be found in individuals with obesity [9, 41]. For example, Son [9] hypothesizes that stability in individuals with obesity is impaired by the response of plantar mechanoreceptors due to increased pressure in the lower extremities. It states that instability in obese individuals is due to reduced foot sensitivity, and that higher instability is compensated by visual in-puts. Villarrasa-Sapina et al. [41] support the theory of Son [9] because they take into the account that obesity in children already attenuates the effects of the somatosensory system for the same reason, namely excessive pressure on the feet. They also add that excessive pressure can affect sensory feedback to such an extent and affect the coordination of body position and the maintenance of postural stability.

Thus, another explanation could be the influence of foot mechanoreceptors on the control of postural stability. The presence of excess body mass can lead to their overload and reduction of the quality of sensory information which is important for control of postural stability. When compared to persons of normal weight, persons with obesity generally have larger plantar contact areas and greater mean pressure values for most anatomical landmarks [42, 43]. For instance, Birtane and Tuna [42] found significantly higher forefoot peak pressure, total plantar force, and total contact area in the feet during static tasks of obese subjects (BMI between 30 and 34.9 kg.m^–2^) when compared to controls. In addition, Hills et al. [44] reported an increase in pressure under the heel, mid-foot, and metatarsal heads II and IV in men with obesity. Glabrous cutaneous receptors in these areas do not display background activity when unloaded [45]. Moreover, mechanoreceptors make a significant contribution to the control and regulation of postural responses, with the frequency of slowly adapting mechanoreceptors rapidly reaching a plateau with increased pressure [46]. This suggests that more significant pressure values and larger contact areas for individuals with obesity may disrupt sensory information resulting from slowly adapting plantar receptors. This could explain why no difference was found between the intervention and control groups, where the change in weight and BMI in post-testing was not significantly different. At the same time, however, according to these claims, weight reduction after bariatric surgery should bring better control of postural stability, which did not happen. It is possible that even greater weight reduction would be required than in our study.

In obese women, higher COP velocities [47, 48] and larger AP ranges [49] have been reported both with and without visual control. This indicates a greater destabilizing impact of body weight on postural control in the sagittal plane. In addition, the study by Menegoni et al. [47] demonstrated the same effects of body weight on postural control, but only with visual control. This is consistent with the results of our study, where a positive correlation of BMI with the COF sway, COF ranges in AP, and COF speed with open eyes was noted. The differences between obese and normal weight can be explained from a biomechanical point of view by accepting the hypothesis that the control postural system can be described using an inverse pendulum model with a pivot point located in the ankle joints [50]. The stability of the human upright position in the sagittal plane is controlled by ankle joint stabilizers [50, 51]. Increased body weight (higher position of center of mass relative to the ankle joint) in individuals with obesity causes an increase in torque at the level of the ankle and consequently an increased need for muscle strength and activity to maintain COP within the base of the support [52, 53]. Because individuals with obesity have lower relative muscle strength (e.g., ankle joint stabilizing muscles related to body weight) than those with normal body mass [54], they have a reduced ability to control sways.

We believe that exercise programs could have a positive effect on faster proprioceptive adaptation to weight loss and changes in body weight distribution. This pilot study did not have enough participants in both groups to completely rule out a positive effect of exercise, so it is therefore possible that exercise can enhance the adaptation process. However, to fully demonstrate any positive effect of exercise, more participants would be necessary and perhaps a longer duration or greater intensity of exercise post-BS may be required.

Another question that emerges from the results is a gender difference in closed eyes setting. In a study by Herrera-Rangel et al. [55], during static posturography, on hard surface, the length of sway was related to peripheral neuropathy, gender, age, and obesity and closing the eyes increased further the difference between genders, which is consistent with the results of the present study. A larger increase of sway after closing the eyes was found in subjects with obesity than in non-obese subjects. This is consistent with Cruz-Gómez et al.’s [56] claim that in individuals with obesity, obesity may be associated with decreased postural stability when vision is not available compared to control and overweight subjects, suggesting that subjects with obesity may be more dependent on vision to control balance. In addition, evidence has shown that after eye closure, the increase in sway in subjects with obesity may be similar when recorded on either a hard or soft surface [56], suggesting that subjects with obesity may use their somatosensation to control posture differently than lean and overweight subjects [55].

Interesting is the relationship of age and some of the postural stability parameters. On one hand, there is positive correlation of the initial total length of the COF sway and age showing decreasing stability during a life span. It is already known as well that sway increases with increasing age [57, 58]. On the other hand, the difference in some parameters was negatively related to age which might show higher chance to improve in postural stability in older subjects.

As for relationship with height, our results are in agreement with other authors who report that with higher height, postural stability deteriorates [7, 59]. The height showed a positive correlation with the initial values of COF sway and speed with the eyes closed. Berger et al. [60] reported that with increasing height, ankle displacement and gastrocnemius response increased.

### Limitations of the study

This study is not without limitations. The small sample size is comparable to past studies in this population, yet a large variability exists in the subjects. Further research involving more participants is needed. Participation in the exercise program was voluntary, possibility creating a healthy-user bias in the exercise portion of the study. It would be more appropriate to select exercising individuals randomly, i.e. to perform a randomized control trial. However, a certain degree of motivation of the individual is required to adhere to the exercise program regime [61] so the participation and exercise adherence could be compromised.

Another possible limitation could have been the relatively short duration (30 sec) of the postural stability test. Another option for future studies would be to repeat the measurement and select only the best result [23]. In our study, no measurement of dynamic postural stability was performed, which would be interesting to include in future research. It would also be appropriate to evaluate the postural stability not only by instrumental posturography, but also with the use of functional stability tests [6, 22].

Another shortcoming of this study can be unavailability of data concerning nutritional habits and possible influence of nutrition on the studied variables.

### Future directions

For further work examining the effect of the exercise program, especially on the change in postural stability, it would be appropriate to compose an exercise program and exercise units which would consist of variety balance exercises, as was the case in the work of Rojhani-Shirazi et al. [22], where a balance exercise program lead to a statistically significant improvement of static and dynamic postural stability; however, stability in their study was not measured by posturographic measurement, but by functional stability tests [22].

Impaired postural stability and damaged balance control are associated with a higher risk of falls [62]. Poor postural stability in combination with higher body weight may result in significant movement restrictions [2, 5]. One way to treat obesity and its associated diseases is to use bariatric-metabolic surgery, which has very good short-term results in the weight reduction. However, a holistic approach with exercise as a cornerstone of treatment is needed in bariatric patients. It is necessary to adopt exercise regime as a key to health. Exercise contributes to maintaining a reduced weight after bariatric surgery, improving quality of life, boosting functional capacity, and increasing cardiorespiratory performance. The full benefits of regular exercise training in obese people who undergone bariatric surgery are various. However, the assumption of improving static postural stability following a three-month exercise program combining aerobic and strength training in the individuals after bariatric surgery cannot be confirmed. Further research including a randomized control trial is needed. It would be also appropriate to create a systematic review or meta-analysis summarizing the results of previous studies dealing with the issue of stability in obese individuals and the possibilities of influencing it.

## Conclusions

Static postural stability did not change in individuals with long-term obesity who underwent bariatric surgery followed by a 3month exercise program. However, we believe that an exercise program should be an integral part of treating patients undergoing bariatric surgery to manage weight, increase weight loss, maintain ideal body weight, and prevent re-gaining weight, as well as to improve cardiometabolic risk factors and quality of life.

## Supporting information

S1 Table Demographic data of the study groups at baseline. (PDF)

S2 Table Description of the strengthening exercise unit parts. (PDF)

S3 Table Mean values (±SD) of weight, BMI, and postural stability parameters in all subjects (N = 22) before and 4 months after bariatric surgery (within group comparison). (PDF)

S4 Table Absolute post-pre differences in BMI and postural stability parameters in the intervention and control group (between groups comparison). (PDF)

S1 Graph Relationship between height and initial values of COF sway-CE. (TIFF)

S2 Graph Relationship between initial BMI and difference in COF sway-OE. (TIFF)

S3 Graph Relationship between age and absolute difference in COF range ML-OE. (TIFF)

S1 File Master data: Before and after BS. (XLSX)

## References

[1] Cawley J. An economy of scales: A selective review of obesity’s economic causes, consequences, and solutions. J Health Econ. 2015;43:244–268. doi: 10.1016/j.jhealeco.2015.03.001 26279519

[2] King WC, Chen JY, Belle SH, et al. Change in Pain and Physical Function Following Bariatric Surgery for Severe Obesity. JAMA. 2016;315(13):1362–1371. doi: 10.1001/jama.2016.3010 27046364 PMC4856477

[3] Gonzalez M, Gates DH, Rosenblatt NJ. The impact of obesity on gait stability in older adults. J Biomech. 2020;100:109585. doi: 10.1016/j.jbiomech.2019.109585 31911052 PMC7061260

[4] Duarte M, Freitas SM. Revision of posturography based on force plate for balance evaluation. Rev Bras Fisioter. 2010;14(3):183–192. 20730361

[5] Vařeka I. Posturální stabilita (I. Část)—terminologie a biomechanické principy. Rehabilitace a fyzikální lékařství. 2002;9(4):115–121.

[6] Horák S, Sovová E, Pastucha D, et al. Comprehensive Group Therapy of Obesity and Its Impact on Selected Anthropometric and Postural Parameters. Cent Eur J Public Health. 2017;25(4):326–331. doi: 10.21101/cejph.a4780 29346858

[7] Hue O, Simoneau M, Marcotte J, et al. Body weight is a strong predictor of postural stability. Gait Posture. 2007;26(1):32–38. doi: 10.1016/j.gaitpost.2006.07.005 16931018

[8] Maffiuletti NA, Agosti F, Proietti M, et al. Postural instability of extremely obese individuals improves after a body weight reduction program entailing specific balance training. J Endocrinol Invest. 2005;28(1):2–7. doi: 10.1007/BF03345521 15816363

[9] Son SM. Influence of Obesity on Postural Stability in Young Adults. Osong Public Health Res Perspect. 2016;7(6):378–381. doi: 10.1016/j.phrp.2016.10.001 28053843 PMC5194219

[10] Villarrasa-Sapiña I, García-Massó X, Serra-Añó P, Garcia-Lucerga C, Gonzalez LM, Lurbe E. Differences in intermittent pos-tural control between normal-weight and obese children. Gait Posture. 2016;49:1–6. doi: 10.1016/j.gaitpost.2016.06.012 27344447

[11] Villarrasa-Sapiña I, Álvarez-Pitti J, Cabeza-Ruiz R, Redón P, Lurbe E, García-Massó X. Relationship between body composition and postural control in prepubertal overweight/obese children: A cross-sectional study. Clin Biomech (Bristol, Avon). 2018;52:1–6. doi: 10.1016/j.clinbiomech.2017.12.010 29291461

[12] Greve J, Alonso A, Bordini AC, Camanho GL. Correlation between body mass index and postural balance. Clinics (Sao Paulo). 2007;62(6):717–720. doi: 10.1590/s1807-59322007000600010 18209913

[13] Błaszczyk JW, Cieślinska-Swider J, Plewa M, Zahorska-Markiewicz B, Markiewicz A. Effects of excessive body weight on postural control. J Biomech. 2009;42(9):1295–1300. doi: 10.1016/j.jbiomech.2009.03.006 19386313

[14] Cieślińska-Świder JM, Błaszczyk JW. Posturographic characteristics of the standing posture and the effects of the treatment of obesity on obese young women. PLoS One. 2019;14(9):e0220962. Published 2019 Sep 4. doi: 10.1371/journal.pone.0220962 31483797 PMC6726190

[15] Teasdale N, Hue O, Marcotte J, et al. Reducing weight increases postural stability in obese and morbid obese men. Int J Obes (Lond). 2007;31(1):153–160. doi: 10.1038/sj.ijo.0803360 16682978

[16] Berrigan F, Simoneau M, Tremblay A, Hue O, Teasdale N. Influence of obesity on accurate and rapid arm movement performed from a standing posture. Int J Obes (Lond). 2006;30(12):1750–1757. doi: 10.1038/sj.ijo.0803342 16619057

[17] Pagnotti GM, Haider A, Yang A, et al. Postural Stability in Obese Preoperative Bariatric Patients Using Static and Dynamic Evaluation. Obes Facts. 2020;13(5):499–513. doi: 10.1159/000509163 33080591 PMC7670358

[18] De Lorenzo A, Soldati L, Sarlo F, Calvani M, Di Lorenzo N, Di Renzo L. New obesity classification criteria as a tool for bariatric surgery indication. World J Gastroenterol. 2016;22(2):681–703. doi: 10.3748/wjg.v22.i2.681 26811617 PMC4716069

[19] Benetti FA, Bacha IL, Garrido AB Junior, Greve JM. Analyses of balance and flexibility of obese patients undergoing bariatric surgery. Clinics (Sao Paulo). 2016;71(2):78–81. doi: 10.6061/clinics/2016(02)05 26934236 PMC4760367

[20] Hue O, Berrigan F, Simoneau M, et al. Muscle force and force control after weight loss in obese and morbidly obese men. Obes Surg. 2008;18(9):1112–1118. doi: 10.1007/s11695-008-9597-5 18584262

[21] Braunerova R, Hainer V. Obesity—diagnostics and treatment in the general practice. Medicine for practice. 2010;7(1):19–22.

[22] Rojhani-Shirazi Z, Azadeh Mansoriyan S, Hosseini SV. The effect of balance training on clinical balance performance in obese patients aged 20–50 years old undergoing sleeve gastrectomy. Eur Surg. 2016;48(2):105–109. doi: 10.1007/s10353-015-0379-8

[23] Zemková E, Kyselovičová O, Jeleň M, et al. Three months of resistance training in overweight and obese individuals improves reactive balance control under unstable conditions. J Back Musculoskelet Rehabil. 2017;30(2):353–362. doi: 10.3233/BMR-160585 27858700

[24] Tekscan. Sportsat 2.0X: User Manual (Rev D). South Boston: Tekscan, 2016.

[25] Tekscan. Pressure Mapping Technology: Pressure Mapping. Force Measurement & Tactile Sensors. https://www.tekscan.com/products-solutions/pressure-mapping-technology. Accessed May 20, 2019.

[26] Bickley C, Linton J, Sullivan E, Mitchell K, Slota G, Barnes D. Comparison of simultaneous static standing balance data on a pressure mat and force plate in typical children and in children with cerebral palsy. Gait Posture. 2019;67:91–98. doi: 10.1016/j.gaitpost.2018.08.012 30308334

[27] Goetschius J, Feger MA, Hertel J, Hart JM. Validating Center-of-Pressure Balance Measurements Using the MatScan^®^ Pressure Mat. J Sport Rehabil. 2018;27(1): doi: 10.1123/jsr.2017-0152 28714837

[28] Thériault-Proulx M, Comtois AS, Murphy N, Boucher JP. Validation of the Matscan^®^ Pressure Mat for Sway Analysis: 1965. Medicine & Science in Sports & Exercise. 2008;40(5). doi: 10.1249/01.mss.0000323377.84898.21

[29] Brenton-Rule A, Mattock J, Carroll M, et al. Reliability of the TekScan MatScan^®^ system for the measurement of postural stability in older people with rheumatoid arthritis. J Foot Ankle Res. 2012;5:21. doi: 10.1186/1757-1146-5-21 22889288 PMC3431264

[30] Cousins SD, Morrison SC, Drechsler WI. Foot loading patterns in normal weight, overweight and obese children aged 7 to 11 years. J Foot Ankle Res. 2013;6(1):36. doi: 10.1186/1757-1146-6-36 23985125 PMC3846107

[31] Tabesh MR, Maleklou F, Ejtehadi F, Alizadeh Z. Nutrition, Physical Activity, and Prescription of Supplements in Pre- and Post-bariatric Surgery Patients: a Practical Guideline [published correction appears in Obes Surg. 2020 Feb;30(2):793]. Obes Surg. 2019;29(10):3385–3400. doi: 10.1007/s11695-019-04112-y 31367987

[32] Handrigan GA, Corbeil P, Simoneau M, Teasdale N. Balance control is altered in obese individuals. J Biomech. 2010;43(2):383–386. doi: 10.1016/j.jbiomech.2009.08.041 19880125

[33] Ruhe A, Fejer R, Walker B. The test-retest reliability of centre of pressure measures in bipedal static task conditions—a systematic review of the literature. Gait Posture. 2010;32(4):436–445. doi: 10.1016/j.gaitpost.2010.09.012 20947353

[34] Raymakers JA, Samson MM, Verhaar HJ. The assessment of body sway and the choice of the stability parameter(s). Gait Posture. 2005;21(1):48–58. doi: 10.1016/j.gaitpost.2003.11.006 15536033

[35] Losco L, Roxo AC, Roxo CW, et al. Helix Thigh Lift. A Novel Approach to Severe Deformities in Massive Weight Loss Patients. J Invest Surg. 2021;1–7. doi: 10.1080/08941939.2021.1912220 34027784

[36] Alonso AC, Luna NM, Mochizuki L, Barbieri F, Santos S, Greve JM. The influence of anthropometric factors on postural balance: the relationship between body composition and posturographic measurements in young adults. Clinics (Sao Paulo). 2012;67(12):1433–1441. doi: 10.6061/clinics/2012(12)14 23295598 PMC3521807

[37] Daniels P, Burns RD, Brusseau TA, et al. Effect of a randomised 12-week resistance training programme on muscular strength, cross-sectional area and muscle quality in women having undergone Roux-en-Y gastric bypass. J Sports Sci. 2018;36(5):529–535. doi: 10.1080/02640414.2017.1322217 28467737

[38] Hassannejad A, Khalaj A, Mansournia MA, Rajabian Tabesh M, Alizadeh Z. The Effect of Aerobic or Aerobic-Strength Exercise on Body Composition and Functional Capacity in Patients with BMI ≥35 after Bariatric Surgery: a Randomized Control Trial. Obes Surg. 2017;27(11):2792–2801. doi: 10.1007/s11695-017-2717-3 28527156

[39] Bankoff ADP, Campelo TS, Ciol P, Zamai CA. Postura e equilíbrio corporal: um estudo das relações existentes. Movimento & Percepção. 2006;6(9):55–70.

[40] Ferreira FPM. Produc¸ão do Journal of Biomechanics entre os anos de 2000 e 2001 relacionada ao tema equilíbrio corporal. [Monografia]. Rio de Janeiro: Universidade do Estado do Rio de Janeiro. 2003.

[41] Villarrasa-Sapiña I, Serra-Añó P, Pardo-Ibáñez A, Gonzalez LM, García-Massó X. Relationship between body composition and vertical ground reaction forces in obese children when walking. Clin Biomech (Bristol, Avon). 2017;41:77–81. doi: 10.1016/j.clinbiomech.2016.12.008 28012303

[42] Birtane M, Tuna H. The evaluation of plantar pressure distribution in obese and non-obese adults. Clin Biomech (Bristol, Avon). 2004;19(10):1055–1059. doi: 10.1016/j.clinbiomech.2004.07.008 15531056

[43] Riddiford-Harland DL, Steele JR, Storlien LH. Does obesity influence foot structure in prepubescent children?. Int J Obes Relat Metab Disord. 2000;24(5):541–544. doi: 10.1038/sj.ijo.0801192 10849573

[44] Hills AP, Hennig EM, McDonald M, Bar-Or O. Plantar pressure differences between obese and non-obese adults: a biome-chanical analysis. Int J Obes Relat Metab Disord. 2001;25(11):1674–1679. doi: 10.1038/sj.ijo.0801785 11753590

[45] Kennedy PM, Inglis JT. Distribution and behaviour of glabrous cutaneous receptors in the human foot sole. J Physiol. 2002;538(Pt 3):995–1002. doi: 10.1113/jphysiol.2001.013087 11826182 PMC2290100

[46] Vedel JP, Roll JP. Response to pressure and vibration of slowly adapting cutaneous mechanoreceptors in the human foot. Neurosci Lett. 1982;34(3):289–294. doi: 10.1016/0304-3940(82)90190-2 6298676

[47] Menegoni F, Galli M, Tacchini E, Vismara L, Cavigioli M, Capodaglio P. Gender-specific effect of obesity on balance. Obesity (Silver Spring). 2009;17(10):1951–1956. doi: 10.1038/oby.2009.82 19325540

[48] Rezaeipour M, Apanasenko G L. Effects of Overweight and Obesity on Postural Stability of Aging Females. Middle East J Rehabil Health Stud. 2018; 5(4):e81617.

[49] Dutil M, Handrigan GA, Corbeil P, et al. The impact of obesity on balance control in community-dwelling older women. Age (Dordr). 2013;35(3):883–890. doi: 10.1007/s11357-012-9386-x 22318311 PMC3636380

[50] Winter DA, Prince F, Frank JS, Powell C, Zabjek KF. Unified theory regarding A/P and M/L balance in quiet stance. J Neuro-physiol. 1996;75(6):2334–2343. doi: 10.1152/jn.1996.75.6.2334 8793746

[51] Maurer C, Peterka RJ. A new interpretation of spontaneous sway measures based on a simple model of human postural control. J Neurophysiol. 2005;93(1):189–200. doi: 10.1152/jn.00221.2004 15331614

[52] Handrigan G, Hue O, Simoneau M, et al. Weight loss and muscular strength affect static balance control. Int J Obes (Lond). 2010;34(5):936–942. doi: 10.1038/ijo.2009.300 20101249

[53] Frankenfield DC, Rowe WA, Cooney RN, Smith JS, Becker D. Limits of body mass index to detect obesity and predict body composition. Nutrition. 2001;17(1):26–30. doi: 10.1016/s0899-9007(00)00471-8 11165884

[54] Wearing SC, Hennig EM, Byrne NM, Steele JR, Hills AP. The biomechanics of restricted movement in adult obesity. Obes Rev. 2006;7(1):13–24. doi: 10.1111/j.1467-789X.2006.00215.x 16436099

[55] Herrera-Rangel A, Aranda-Moreno C, Mantilla-Ochoa T, Zainos-Saucedo L, Jáuregui-Renaud K. The influence of peripheral neuropathy, gender, and obesity on the postural stability of patients with type 2 diabetes mellitus. J Diabetes Res. 2014;2014:787202. doi: 10.1155/2014/787202 25258716 PMC4167211

[56] Cruz-Gómez NS, Plascencia G, Villanueva-Padrón LA, Jáuregui-Renaud K. Influence of obesity and gender on the postural stability during upright stance. Obes Facts. 2011;4(3):212–217. doi: 10.1159/000329408 21701237 PMC6444809

[57] Era P, Sainio P, Koskinen S, Haavisto P, Vaara M, Aromaa A. Postural balance in a random sample of 7,979 subjects aged 30 years and over. Gerontology. 2006;52(4):204–213. doi: 10.1159/000093652 16849863

[58] Røgind H, Lykkegaard JJ, Bliddal H, Danneskiold-Samsøe B. Postural sway in normal subjects aged 20–70 years. Clin Physiol Funct Imaging. 2003;23(3):171–176. doi: 10.1046/j.1475-097x.2003.00492.x 12752561

[59] Fabunmi AA, Gbiri CA. Relationship between balance performance in the elderly and some anthropometric variables. Afr J Med Med Sci. 2008;37(4):321–326. 19301708

[60] Berger W, Trippel M, Discher M, Dietz V. Influence of subjects’ height on the stabilization of posture. Acta Otolaryngol. 1992;112(1):22–30. doi: 10.3109/00016489209100778 1575033

[61] Vartanian LR, Shaprow JG. Effects of weight stigma on exercise motivation and behavior: a preliminary investigation among college-aged females. J Health Psychol. 2008;13(1):131–138. doi: 10.1177/1359105307084318 18086724

[62] Matrangola SL, Madigan ML. Relative effects of weight loss and strength training on balance recovery. Med Sci Sports Exerc. 2009;41(7):1488–1493. doi: 10.1249/MSS.0b013e31819bd4bd 19516151

