# Correction: Bariatric surgery and exercise: A pilot study on postural stability in obese individuals

**DOI:** 10.1371/journal.pone.0296558

**Published:** 2023-12-28

**Authors:** Natálie Cibulková, Klára Daďová, Kateřina Mašková, Andrew Busch, Alena Kobesová, Jitka Vařeková, Marcela Hašpicová, Martin Matoulek

There are errors in the Funding section. The correct Funding statement is: This study was supported by the Ministry of Health, Czech Republic (GJIH-1599-04-1-180)—conceptual development of research organization 64165, General University Hospital in Prague, Czech Republic, the Program of the institutional support for science at Charles University Progress, No. Q41 Biological aspects of the investigation of human movement, and the Foundation Movement without Help. The funders had no role in study design, data collection and analysis, decision to publish, or preparation of the manuscript.
