# Supplementary material for: Bariatric surgery and exercise: A pilot study on postural stability in obese individuals
Source: PLoS One. 2022 Jan 14;17(1):e0262651. doi: 10.1371/journal.pone.0262651 (PMC8759698; doi:10.1371/journal.pone.0262651)
Supplement: S1 Table — (PDF) [file pone.0262651.s001.pdf]

Table 1. Demographic data of the study groups at baseline

	**Exercising group (EX)**	**Non-exercising group (NEX)**	p-Value
Number [n (women; men)]	10 (4;6)	12 (4;8)	
Age (years)	48.9 ± 7.5	44.7 ± 13.6	0.389
Height (m)	1.75 ± 0.13	1.75 ± 0.10	0.847
Weight (kg)	128 ± 23.1	131 ± 22.8	0.737
BMI ($kg.m^{-2}$)	42 ± 5.6	42.6 ± 6.0	0.825

Note: Values are expressed as means± SD
