# Supplementary material for: Bariatric surgery and exercise: A pilot study on postural stability in obese individuals
Source: PLoS One. 2022 Jan 14;17(1):e0262651. doi: 10.1371/journal.pone.0262651 (PMC8759698; doi:10.1371/journal.pone.0262651)
Supplement: S2 Table — (PDF) [file pone.0262651.s002.pdf]

Table 2. Description of the strengthening exercise unit parts

Training Segment	Length (min)	Description
Warm-up	10-15	Total warming up and preparation using aerobic devices
Preparation	10	Preparation of muscle and joint structures using circular and pendulum movements, respiratory physiotherapy to prepare the respiratory system for exercise
Resistance Exercises	30-35	Strengthening the muscles of the whole body, training stability, stabilization, support
Cool-down	10-15	Soothing exercises with mainly stretching exercises and respiratory exercises to calm down
