# Supplementary material for: Bariatric surgery and exercise: A pilot study on postural stability in obese individuals
Source: PLoS One. 2022 Jan 14;17(1):e0262651. doi: 10.1371/journal.pone.0262651 (PMC8759698; doi:10.1371/journal.pone.0262651)
Supplement: S3 Table — (PDF) [file pone.0262651.s003.pdf]

Table 3. Mean values (±SD) of weight, BMI, and postural stability parameters in all subjects (N=22) before and 4 months after bariatric surgery (within group comparison)

		Before BS	After BS	p-Value
**Weight (kg)**		131.4±22.5	113.2±19.8	<0.001
**BMI ($kg.m^{-2}$)**		42.6±6.0	36.7±4.9	<0.001
**COF sway (cm)**	OE	28.28±10.96	27.89±10.74	0.684
	CE	45.70±18.75	47.55±23.25	0.372
**Range AP (cm)**	OE	1.96±0.79	2.18±0.74	0.108
	CE	2.89±0.83	2.93±1.07	0.961
**Range ML (cm)**	OE	1.76±0.94	1.87±0.80	0.498
	CE	2.06±1.21	2.31±1.36	0.306
**COF speed (cm/s)**	OE	0.94±0.37	0.93±0.36	0.697
	CE	1.53±0.63	1.59±0.78	0.372

Note: COF – center of force, OE – open eyes, CE – closed eyes, SD – standard deviation
