# Supplementary material for: Bariatric surgery and exercise: A pilot study on postural stability in obese individuals
Source: PLoS One. 2022 Jan 14;17(1):e0262651. doi: 10.1371/journal.pone.0262651 (PMC8759698; doi:10.1371/journal.pone.0262651)
Supplement: S4 Table — (PDF) [file pone.0262651.s004.pdf]

Table 4. Absolute post-pre differences in BMI and postural stability parameters in the Intervention and Control group (between groups comparison)

Post-pre difference (mean ± SD)		Control (NEX)	Intervention (EX)	p-Value
**BMI ($kg.m^{-2}$)**		-5.46	-6.58	0.621
**COF sway (cm)**	OE	-0.65	-0.76	0.717
	CE	-0.23	1.90	0.869
**Range AP (cm)**	OE	0.09	0.27	0.717
	CE	-0.21	0.32	0.199
**Range ML (cm)**	OE	-0.08	0.16	0.598
	CE	0.24	0.07	0.575
**COF speed (cm/s)**	OE	-0.02	-0.02	0.742
	CE	-0.01	0.06	0.869

Note: COF – center of force, OE – open eyes, CE – closed eyes, SD – standard deviation
