# Supplementary figures and images for: Bariatric surgery and exercise: A pilot study on postural stability in obese individuals
Source: PLoS One. 2022 Jan 14;17(1):e0262651. doi: 10.1371/journal.pone.0262651 (PMC8759698; doi:10.1371/journal.pone.0262651)

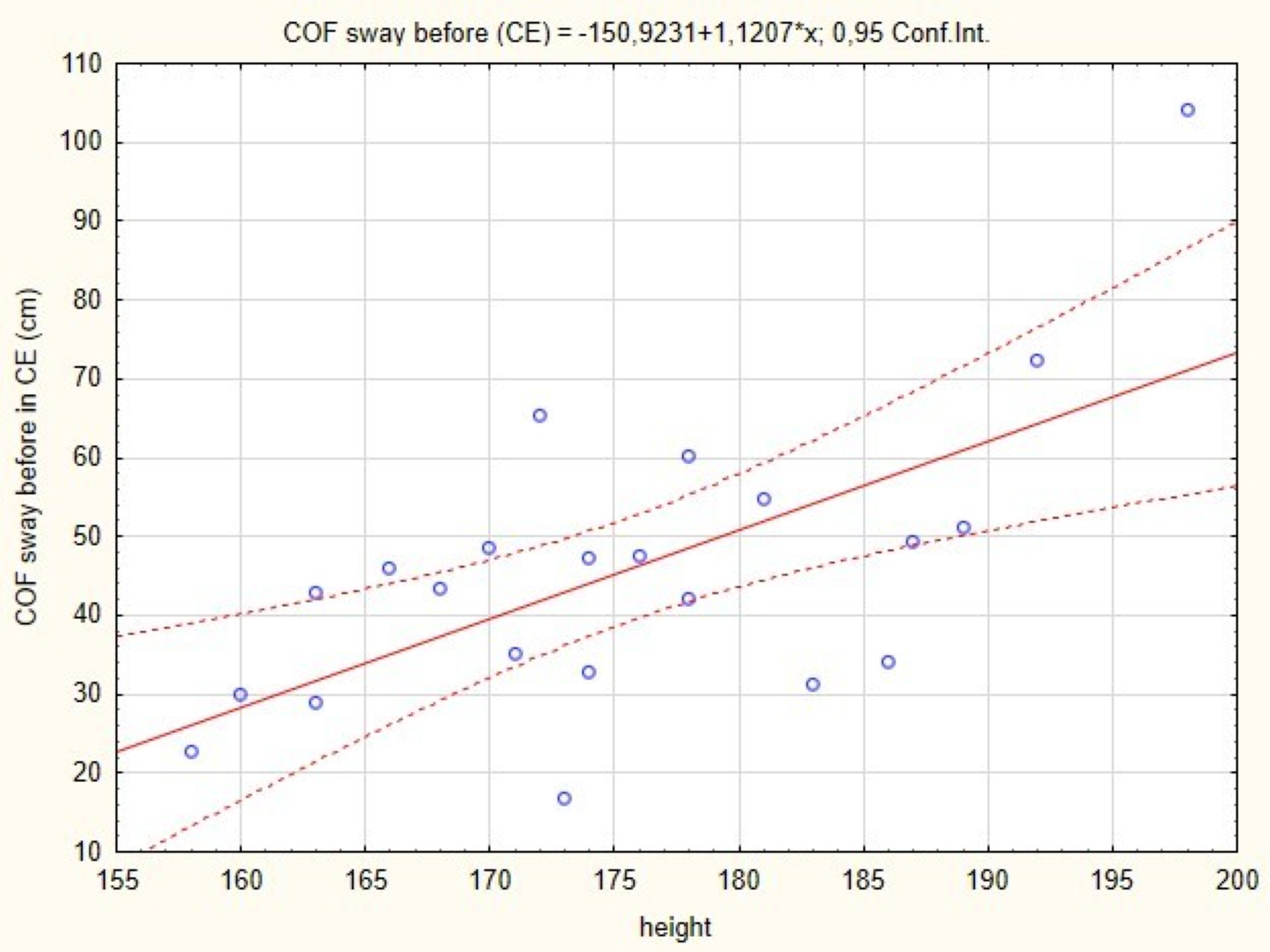

Supplement: S1 Graph — (TIFF) [file pone.0262651.s005.tiff]

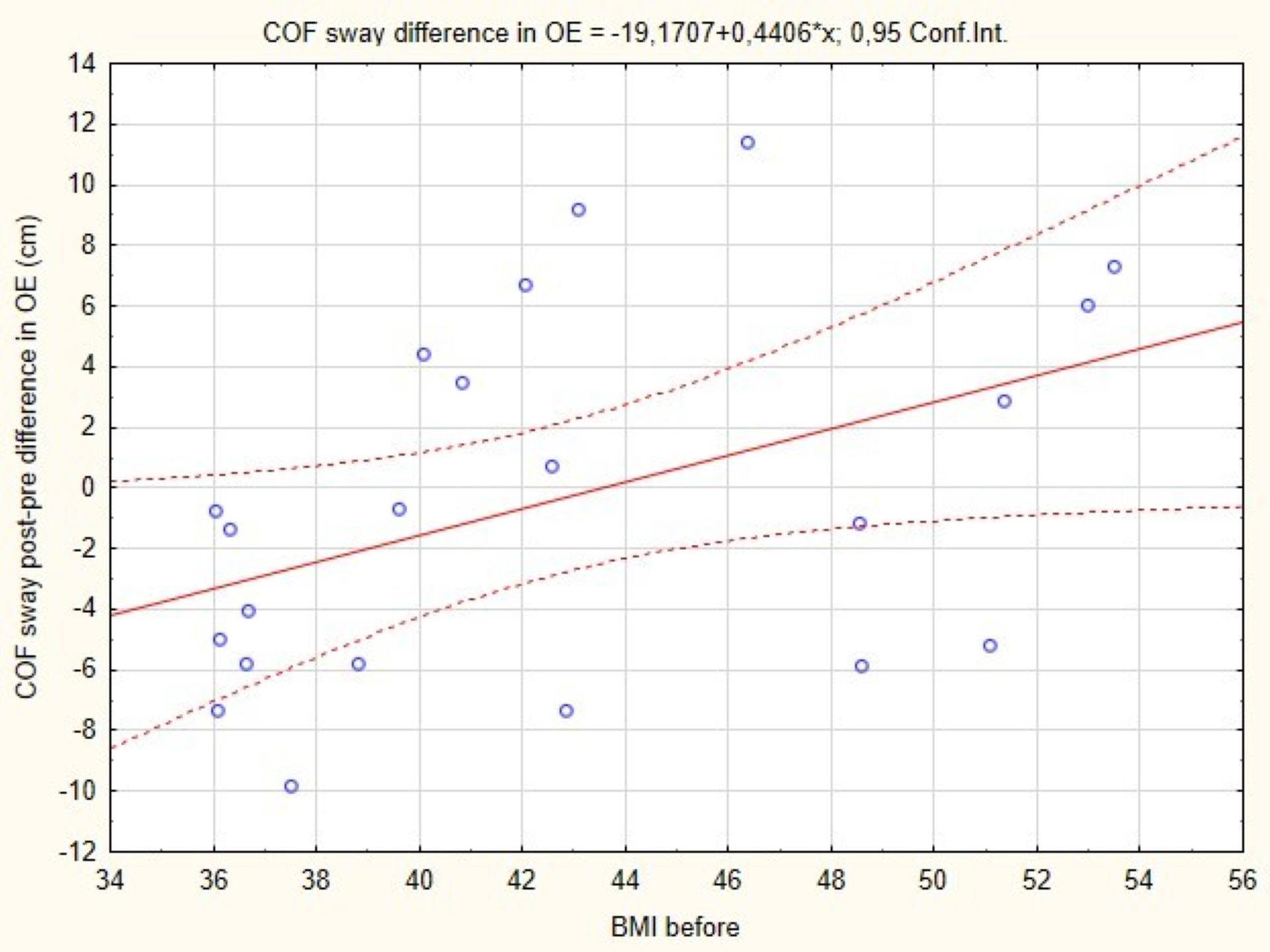

Supplement: S2 Graph — (TIFF) [file pone.0262651.s006.tiff]

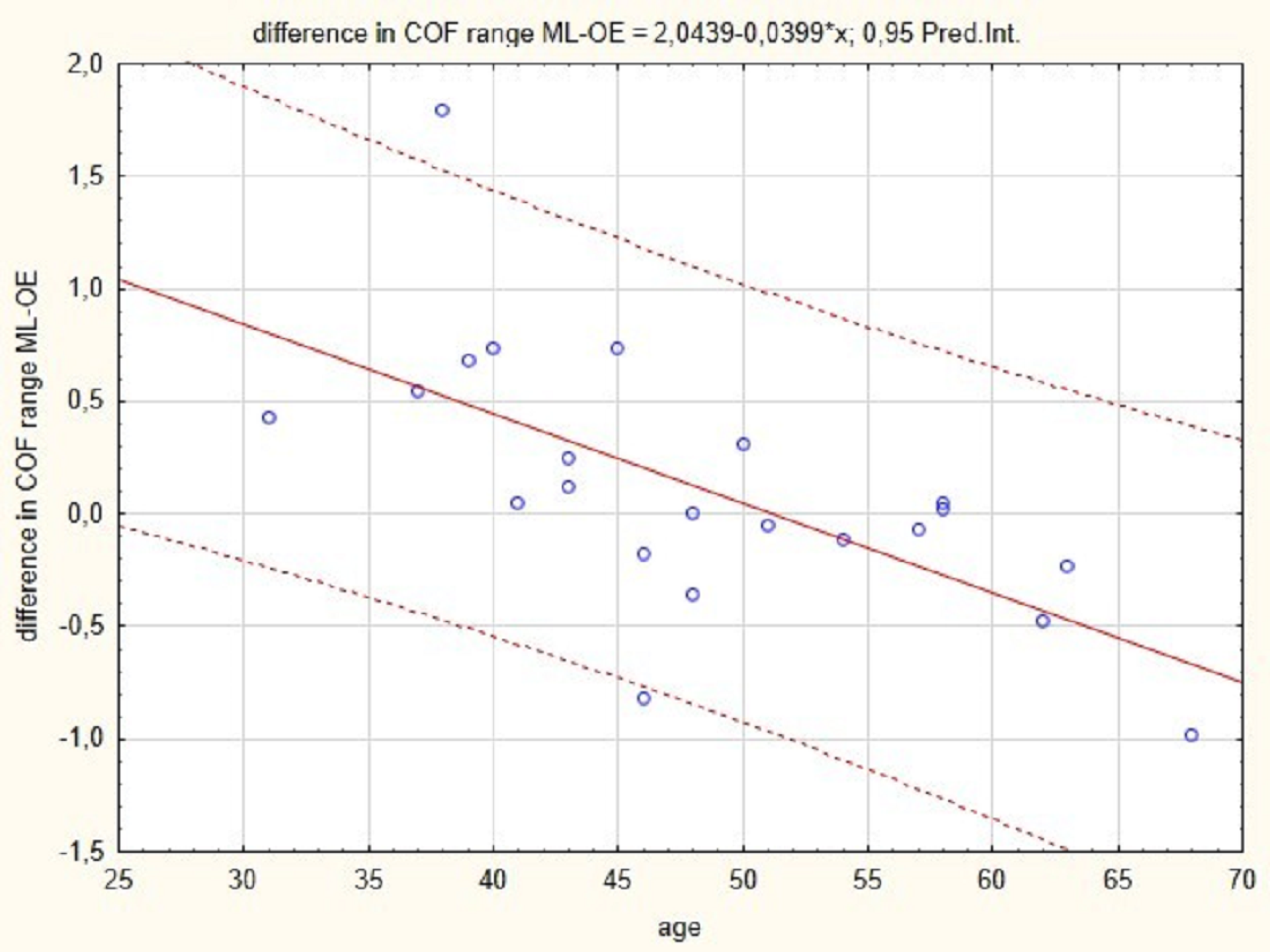

Supplement: S3 Graph — (TIFF) [file pone.0262651.s007.tiff]
